# Inhalational sevoflurane in severe bronchial obstruction unresponsive to multipharmacologic therapy: a case report

**DOI:** 10.12688/f1000research.1-56.v1

**Published:** 2012-11-26

**Authors:** Thomas Weber, Christian Schiebenpflug, Engelbert Deusch

**Affiliations:** 1 Department of Anaesthesiology and Intensive Care, Sozialmedizinisches Zentrum Ost-Donauspital, Langobardenstr. 122, Vienna, Austria; 2 Department of Anaesthesiology and Intensive Care, Otto-Wagner-Spital, Sanatoriumsstrasse 1, 1140 Vienna, Austria

## Abstract

**Introduction:** Bronchial asthma with respiratory failure is a challenge for the intensivist as mechanical ventilation is often difficult due to bronchoconstriction and air-trapping. We describe a case of severe asthma with respiratory acidosis in a 10-year-old patient unresponsive to multipharmacologic broncholytic therapy. Only the initiation of sevoflurane inhalation resolved severe bronchoconstriction and dynamic hyperinflation, leading to complete recovery.

**Case presentation:** A 10-year-old Caucasian boy was intubated and mechanically ventilated due to an asthmatic attack. Bronchoconstriction and dynamic hyperinflation were severe while multipharmacological broncholytic therapy was unsuccessful. Inhalation with sevoflurane via an anaesthesia machine was the key intervention leading to gradual resolving of severe hypercapnia and respiratory acidosis. Furthermore bilateral pupil dilation occurred during hypercapnia, but no intracranial pathology could be detected. The patient made an uneventful recovery. To our knowledge this is the first case where hypercapnia and respiratory acidosis were so profound and long lasting yet the patient survived without any damage.

**Conclusions:** Inhalational anaesthetics must be considered as an early treatment option in ventilated asthmatic patients with bronchial obstruction unresponsive to conventional therapy even though their administration in intensive care units may be difficult.

## Case description

A 10 year-old boy weighing 40 kilograms was admitted to the Paediatric Intensive Care Unit after being found unconscious at home, and was subsequently intubated by an emergency care team. He had a history of asthma starting at the age of 4. Asthmatic episodes in the past were treated with intermittent salbutamol disc inhaler applications.

**Figure 1.**
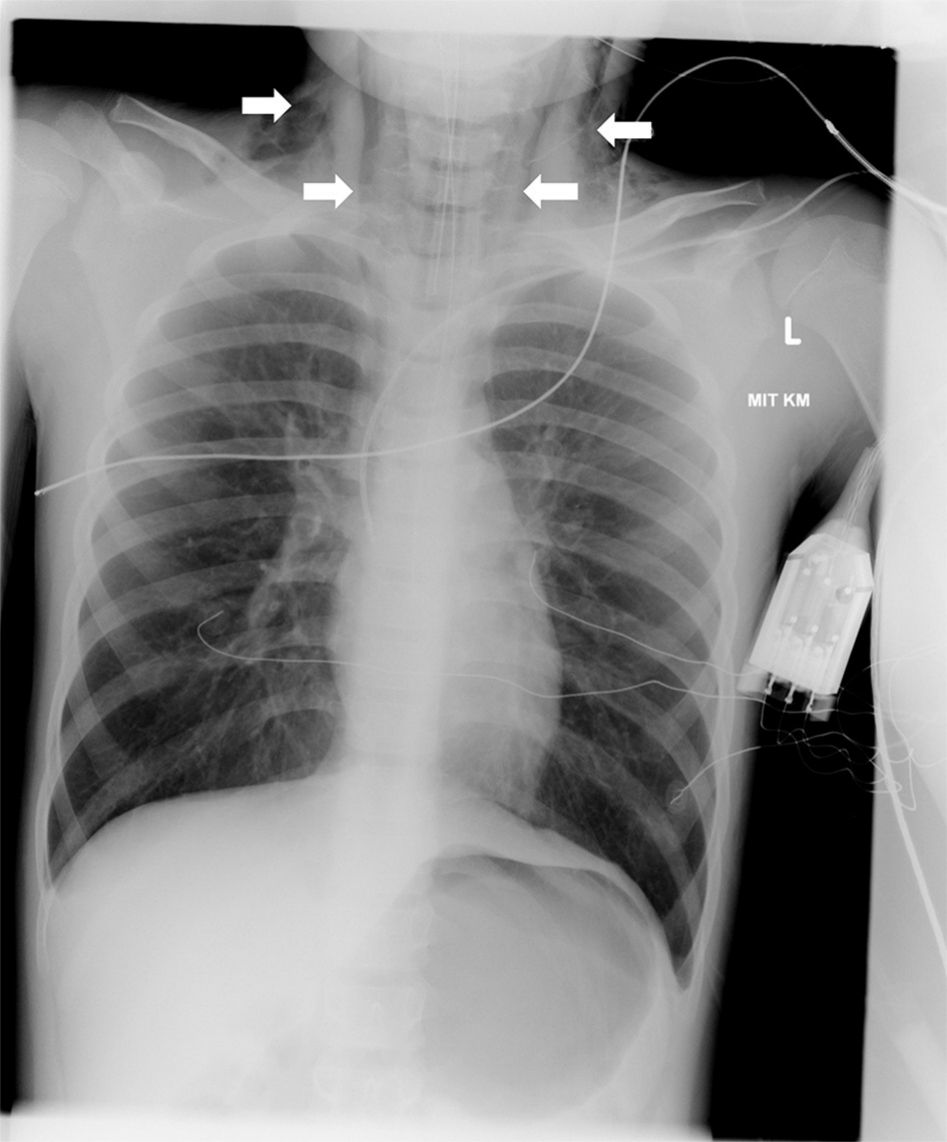
Chest X-ray showing hyperinflated lungs and a discrete subcutaneous emphysema at the neck and the upper mediastinum (arrows).

On admission the patient had a plethysmographic oxygen saturation of 80% on mechanical ventilation with an inspired oxygen fraction of 100%. Initial blood gas analysis (ABL 700 Radiometer Copenhagen) revealed a paCO _2_ (partial pressure of arterial carbon dioxide) of >256 mm Hg, which was exceeding the cut-off-level of the analyser, and a pH of 6.69. The initial chest X-ray showed hyperinflated lungs and a discrete subcutaneous emphysema at the neck and the upper mediastinum ( Figure 1). The patient was sedated with fentanyl 4 µg/kg/h, midazolam 0.5 mg/kg/h and ketamine 2 mg/kg/h and ventilated with a Draeger Evita 4 respirator (Draeger, Luebeck, Germany) using a pressure controlled mode. Initial ventilator settings were plateau pressure 36 cm H _2_O, positive endexspiratory pressure (PEEP) 5 cm H _2_O, respiratory rate 12/min and I/E ratio 1:3 with 100% inspired oxygen (ratio of inspiration to expiration in mechanical ventilation). This achieved sufficient oxygenation, but the flow pattern display on the respirator revealed massive air-trapping.

Broncholytic therapy was started with prednisolone 2 mg/kg, terbutaline 0.02 mg/kg/h i.v., magnesium sulphate 2 g over 20 min i.v. q6h and inhalation with salbutamol and ipratropium bromide q4h. An antibiotic treatment with amoxicillin/clavulanic acid 1.5 g q8h and clarithromycin 300 mg q12h was initiated.

**Figure 2.**
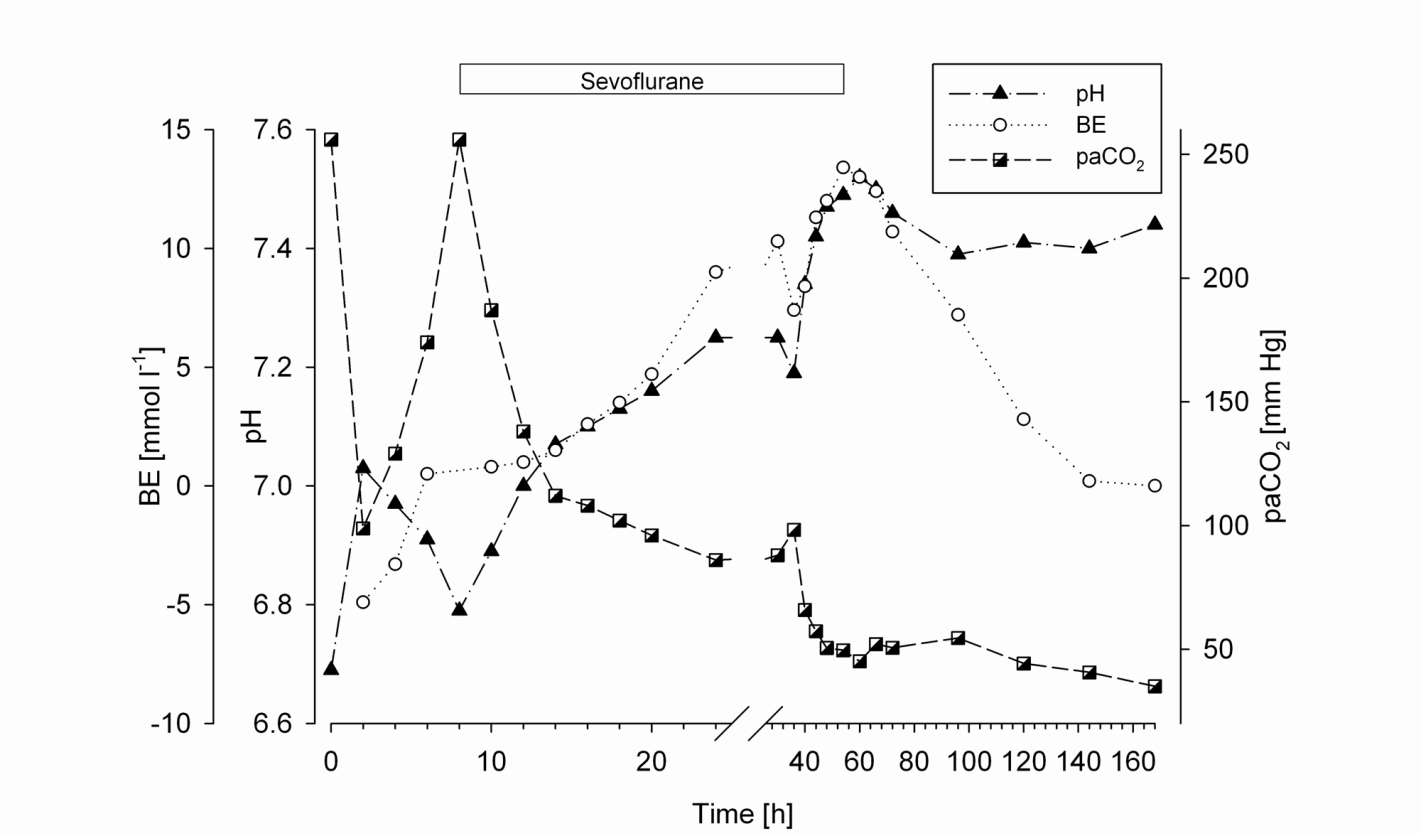
Base excess (BE) and pH are shown on the left side of the y-axis. Partial pressure of arterial CO _2_(paCO _2_) is shown on the right side of the y-axis. Time course in hours (h) is shown on the x-axis.

The ventilation management proved to be difficult in this boy. Ventilator settings had to be increased in a stepwise mode to plateau pressures up to 45 cm H _2_O and PEEP reduced to 0 cm H _2_O in the first two hours. However, neither severe hypercapnia nor air-trapping improved. Then, a combined high and low-frequency ventilation was initiated (VDR4 percussion ventilator, Reiner, Germany) with a percussion frequency of 400/min and a conventional frequency of 10/min. With this regime, paCO _2_ could be reduced to 98 mm Hg and pH raised to 7.03. Unfortunately hypercapnia worsened again and mediastinal emphysema was more prominent. 10 h after admission, the blood gas analysis revealed a paCO _2_ of >256 mmHg and a pH of 6.79. The respirator was replaced by an anaesthesia machine (Draeger Julian, Luebeck, Germany) and inhalation of 3% sevoflurane was started. Within minutes tidal volume increased from 100 to 320 ml while the plateau pressure could be reduced from 43 to 35 cm H _2_O. Other respiratory settings were PEEP 0, respiratory rate 6/min, I:E 1:5 and inspired oxygen fraction 100%. From that time blood gases improved continuously and air-trapping decreased with a paCO _2_ falling below 100 mm Hg after 20 hours and a pH exceeding 7.2 after 24 hours. Oxygen fraction could then be reduced to 50%. No buffering was performed throughout the whole treatment. Because blood pressure levels tended to be low (MAP < 60 mm Hg) despite a positive fluid balance, Dobutamine 4 µg/kg/min and Norepinephrine 0.08 µg/kg/min i.v. were administered. The time course of the patient´s blood gases is shown graphically in Figure 2.

15 h after admission a fixed dilation of both pupils was observed. A cerebral CT-scan showed no abnormalities like brain swelling or intracranial bleeding. Since there was a risk of a longer hypoxic period, cooling to a core temperature of 34°C was initiated (Arctic Sun cooling system, Medivance, CO, USA) and intracranial pressure monitoring was performed (Codman® ICP monitoring system) revealing normal values.

After 36 h, the situation had significantly improved: sevoflurane, terbutaline and ipratropium bromide could be stopped, magnesium sulphate was reduced and the anaesthesia machine could be replaced by an intensive care respirator. Chest X-rays showed that the subcutaneous and mediastinal emphysema had resolved. Subsequently, magnesium infusions were stopped and prednisolone was tapered. After 48 h, pupil dilation slowly resolved and 2 further days later pupils showed intact light reaction. Catecholamines were stopped and sedation was gradually weaned. On day 5 spontaneous breathing started, intracranial pressure monitoring was terminated and the trachea was extubated after 8 days. The boy was transferred to the regular ward on day 10 without any neurologic impairment and could be discharged in good condition 4 days later.

## Discussion

We report a young patient suffering from a severe asthmatic attack that only resolved after therapy with inhalational anaesthesia using sevoflurane. During the treatment period prolonged severe hypercapnia and respiratory acidosis was observed. Moreover, the patient developed pupil dilatation that persisted for more than 30 hours. However, therapy was successful and the patient recovered completely.

To our knowledge the duration of hypercapnia with a peak paCO _2_ >256 mmHg, a paCO _2_ >100 mmHg for 20h and respiratory acidosis with a pH less than 7.2 for 24 h associated with a complete recovery without any complications has not been reported so far. In a case of an asthmatic patient described by Mazzeo and colleagues, where peak paCO _2_ level was 293 mmHg and pH 6.77 ^1^, hypercapnia and respiratory acidosis resolved approximately 12 h after onset.

In our patient, pharmacological therapy with different topic and intravenous broncholytic agents failed. Ventilation management was complicated whereas adequate oxygenation could be achieved without major problems.

The major risk of massive bronchospasm with consecutive air-trapping is pulmonary hyperinflation leading to barotrauma and, on the other hand, increased pulmonary vascular resistance resulting in right ventricular failure. Therefore, low tidal volumes, a low respiratory frequency and a low I:E ratio are recommended strategies, whereas application of external PEEP remains a controversial issue ^2, 3^. The goal is to achieve sufficient oxygenation and a reduction of hypercapnia. Several animal studies showed that even high levels of paCO _2_ and respiratory acidosis can be well tolerated ^4, 5^ whereas buffering respiratory acidosis was found to worsen lung injury ^6^.

In our patient a trial with high-frequency ventilation to facilitate CO _2_ elimination was initially successful, but subsequently resulted in deterioration of ventilation parameters.

A multipharmacologic approach was used combining i.v. corticosteroids (prednisolone), i.v. and inhaled β-adrenergic agents (terbutaline and salbutamol), inhaled anticholingergic agents (ipratropium bromide) and i.v. magnesium and ketamine. All these agents influence bronchial tone by different mechanisms and our goal was to achieve a synergistic effect. However, the key therapeutic intervention for resolving airway obstruction was inhalational anaesthesia with sevoflurane. In the aforementioned case, different broncholytic agents and sevoflurane inhalation were applied but it remained questionable which agent was most effective ^1^. Sevoflurane is known to modulate bronchial tone via voltage-dependent Ca ^++^-channel activity and intracellular cyclic adenosine monophosphate levels ^7^. Among anaesthetists sevoflurane inhalation is common practice in the theatre today in cases of bronchial obstruction after tracheal intubation. However, in the majority of intensive care settings, sevoflurane cannot be easily applied since most intensive care respirators are not designed to administer volatile anaesthetics. Getting an anaesthesia respirator to the ICU and changing the machine is cumbersome and may put the patient at further risk. Recently a new rebreathing device for the application of volatile anaesthetics in the ICU has become available (AnaConDa, Sedana Medical, Sundbyberg, Sweden), that allows wash-in kinetics for sevoflurane comparable to a regular vaporizer ^8^.

Extracorporal CO _2_ elimination can be considered another treatment option to remove hypercapnia and respiratory acidosis, and a pumpless arterio-venous system has been recently used for treatment in children ^9^.

The clinical course of our patient was further complicated by bilateral dilated pupils. The findings could be explained by the occurrence of intracranial pathologies. Indeed, there are some case reports in ventilated asthmatic patients where permissive hypercapnia resulted in intracranial hypertension and even subarachnoidal haemorrhage ^10– 13^. In our patient however, CT scan was unremarkable. Moreover, contamination with inhaled anticholinergic drugs (like ipratropiumbromide) has also been blamed for unilateral pupillary dilation ^14^, however, in our patient, symmetrical abnormalities were observed, the patient was ventilated before arrival on the ICU and had eye protection while receiving broncholytic therapy. Due to a potentially prolonged hypoxic event, systemic cooling therapy and continuously invasive intracranial pressure monitoring was performed for 48 h, but unfortunately the cause of symmetrical pupil dilation remains unclear.

## Summary

Inhalational anaesthetics should be considered as an early treatment option in ventilated asthmatic patients with unresponsive bronchial obstruction.

## Consent

Written informed consent for publication was obtained from the patient`s parents for publication of this case report and accompanying images.

## References

[1] Mazzeo AT Spada A Pratico C: Hypercapnia: what is the limit in pediatric patients? A case of near-fatal asthma successfully treated by multipharmacological approach. *Pediatr Anesth.* 2004;14(7):596–603 10.1111/j.1460-9592.2004.01260.x 15200659

[2] Brenner B Corbridge T Kazzi A: Intubation and mechanical ventilation of the asthmatic patient in respiratory failure. *J Allergy Clin Immunol.* 2009;124(2 Suppl):S19–28 10.1016/j.jaci.2009.05.008 19647132

[3] Jain S Hanania NA Guntupalli K: Ventilation of Patients with asthma and obstructive lung disease. *Crit Care Clin.* 1998;14(4):685–705 10.1016/S0749-0704(05)70026-9 9891633

[4] Vannucci RC Towfighi J Heitjan DF: Carbon dioxide protects the perinatal brain from hypoxic-ischemic damage: an experimental study in the immature rat. *Pediatrics.* 1995;95(6):868–874 7761212

[5] Litt L Gonzalez-Mendez R Severinghaus JW: Cerebral intracellular changes during supercarbia: an in vivo 31P nuclear magnetic resonance study in rats. *J Cereb Blood Flow Metab.* 1985;5(4):537–544 10.1038/jcbfm.1985.81 4055925

[6] Nichol AD O´Cronin DF Howell K: Infection-induced lung injury is worsened after renal buffering of hypercapnic acidosis. *Crit Care Med.* 2009;37(11):2953–2561 10.1097/CCM.0b013e3181b028ce 19773647

[7] Iwasaki S Yamakage M Satoh J: Different inhibitory effects of sevoflurane on hyperreactive airway smooth muscle contractility in ovalbumin-sensitized and chronic cigarette-smoking guinea pig models. *Anesthesiology.* 2006;105(4):753–763 17006075 10.1097/00000542-200610000-00022

[8] Sturesson LW Johansson A Bodelsson M: Wash-in kinetics for sevoflurane using a disposable delivery system (AnaConDa) in cardiac surgery patients. *Br J Anaesth.* 2009;102(4):470–476 10.1093/bja/aep019 19244261

[9] Conrad SA Green R Scott LK: Near-fatal pediatric asthma managed with pumpless arteriovenous carbon dioxide removal. *Crit Care Med.* 2007;35(11):2624–2629 10.1097/01.CCM.0000288104.97602.B3 17901835

[10] Gaussorgues P Piperno D Fouque F: Hypertension intracranienne au cours de l’etat de mal asthmatique.Article in French. *Ann Fr Anesth Reanim.* 1987;6(1):38–41 10.1016/S0750-7658(87)80008-4 3578944

[11] Dimond JP Palazzo MG: An unconscious man with asthma and a fixed, dilated pupil. *Lancet.* 1997;349(9045):98 10.1016/S0140-6736(96)10507-9 8996423

[12] Rodrigo R Rodrigo G: Subarachnoid hemorrhage following permissive hypercapnia in a patient with severe acute asthma. *Am J Emerg Med.* 1999;17(7):697–699 10.1016/S0735-6757(99)90164-X 10597094

[13] Edmunds SM MD Harrison R: Subarachnoid hemorrhage in a child with status asthmaticus: Significance of permissive hypercapnia. *Pediatr Crit Care Med.* 2003;4(1):100–103 10.1097/01.PCC.0000031369.98596.A6 12656553

[14] Udy A: A 10-year-old child with status asthmaticus, hypercapnia and a unilateral dilated pupil. *Pediatr Anesth.* 2005;15(12):1120–1123 10.1111/j.1460-9592.2005.01581.x 16324036

