# Supplementary material for: Inhalational sevoflurane in severe bronchial obstruction unresponsive to multipharmacologic therapy: a case report
Source: F1000Res. 2012 Nov 26;1:56. [Version 1] doi: 10.12688/f1000research.1-56.v1 (PMC3790599; doi:10.12688/f1000research.1-56.v1)
Supplement: Peer review history

# Peer review: Referee response for version 1

**Published:** 2012-12-18

**Authors:** Robin Steinhorn

**Affiliations:** 1 Department of Pediatrics, University of California Davis Children's Hospital, Sacremento, California, USA

This is an interesting case report of a child who presented with profound acidosis due to an asthma exacerbation.

While the idea of terminating asthma with inhaled isoflurane or sevoflurane has been reported in the literature, this case report is notable for its severity, use in conjunction with core cooling, and good outcome.

I have read this submission. I believe that I have an appropriate level of expertise to confirm that it is of an acceptable scientific standard.

# Peer review: Referee response for version 1

**Published:** 2012-12-10

**Authors:** Peter Nagele

**Affiliations:** 1 School of Medicine, Department of Anesthesiology, Washington University in St Louis, St Louis, MO, USA

I have read this submission. I believe that I have an appropriate level of expertise to confirm that it is of an acceptable scientific standard.

# Peer review: Referee response for version 1

**Published:** 2012-11-26

**Authors:** Cesar Picado

**Affiliations:** 1 Hospital Clínic de Barcelona, Institut de Pneumologia, University of Barcelona, Barcelona, Spain

This is an interesting case report. Currently, there is very limited information regarding the treatment of severe asthma exacerbation which is difficult-to treat and doesn’t responding to conventional therapy. The approach described in this case report suggests an alternative treatment that could contribute to saving the lives of asthma patients.

I have read this submission. I believe that I have an appropriate level of expertise to confirm that it is of an acceptable scientific standard.
